# Supplementary material for: Identification and Validation of Esophageal Squamous Cell Carcinoma Targets for Fluorescence Molecular Endoscopy
Source: Int J Mol Sci. 2021 Aug 27;22(17):9270. doi: 10.3390/ijms22179270 (PMC8431213; doi:10.3390/ijms22179270)

Supplemental tables

Supplementary Table S1 *Summary of microarray expression profiles of ESCC and normal esophagus for FGmRNA profiling.*

**Data source**	**Sample size of normal esophageal squamous epithelium**	**Sample size of ESCC**
Kimchi ET et al., Cancer Research 2005. [37]	8	0
Stairs DB et al., Plos One 2008. [38]	7	0
Wang Q et al., BMC Cancer 2013. [39]	19	9
Lee JJ et al., Carcinogenesis 2010. [40]	5	5
Yu K et al., Plos Genetics 2008. [41]	13	16

Hyland PL et al., International Journal of Epidemiology 2016. [42]	53	53
Aoyagi K et al., Plos One 2011. [43]	0	20
Chen K et al., Plos One 2013. [44]	15	15
**Total**	120	118

Supplementary Table S3 *Literature search results of top 60 genes*

**RANK**	**GENE**	**Location**	**Location (genecards)**	**Location (ICAN)**	**IHC esophagus**	**IHC GE**	**Carcinogenesis**	**Imaging**	**Ref.**
1	PSMD2	IC,M	N, cytosol, EC	cytosolic proteasome complex, DNA directed RNA polymerase III complex	None	None	Matsuyama Y,et al [45].	None	[45]
2	GREM1	S	EC	Proteinaceous extracellul	None	None	Davis H,et al [46]; van Vlodrop	None	[46-47

				ar matrix			IJ,et al [47].		]
3	BMP 7	IC,S	EC	Extracellular matrix, synaptic membrane	Xu G, et al [48]	Motoyama K, et al. [49]	Alarmo EL, et al [50].	None	[48-50]
4	XCL 1	S	EC	Not assigned	None	None	Wang T, et al. [51]	None	[51]
5	SLC33A1	M	M;endoplasmic reticulum; golgi apparatus	Side of membrane	None	None	None	None	
6	XCL 2	S	EC	Not available	None	None	Kim M, et al [52]	None	[52]
7	SERP 1	IC,M	Cytoskeleton; cytosol; endoplasmic reticulum	Not assigned	None	None	Not conclusive	None	
8	GSTP 1	IC	Cytosol, EC, mitochondrion	Not assigned	Yamamoto Y, et al. [54] Allameh A,et al [55].	Zhang R, et al [56].	Louie SM, et al. [53]	Fujikawa Y, et al [57].	[53-57]
9	FXR1	IC,S	N,cytosol	CUL3 ring ubiquitin ligase complex	None	Jin X, et al. [58]	Qian J, et al. [59]	None	[58-59]
10	SPRR1B/cornifin B	IC	EC,M, cytosol	Cornified envelope, keratin filament	None.	None	Michifuri Y, et al [60].	None	[60]
11	COPB2	IC	Cytosol;golgi apparatus ;ER	COPI coated vesicle	None	None	Pu X, et al [61].	None	[61]

				membrane, golgi associated vesicle membrane					
12	FZD6	M	M	Apicolateral plasma membrane	None	Kim BK, et al [62].	Yan J, et al [63].	None	[62-63]
13	NDUFB5	IC,M,S	Mitochondrion;nucleus	Oxidoreductase complex, mitochondrial membrane part, cytochrome complex	None	None	None	None	
14	HOXD10	IC	Nucleus,cytosol	Proteinaceous extracellular matrix	None	None	Wang L, et al [64].	None	[64]
15	HOXD11	IC	nucleus	Ionotropic gutamate receptor compelx	None	None	Not conclusive	None	
16	PKP1	IC	Cytoskeleton; EC;N;M	Intermediate filament, desmosome, cell cell junction, anchoring junction, cornified envelope	None	None	Lee P, et al. [65]	None	[65]

17	HSP90AB1	IC	Cytosol;EC;N; mitochondrion	Ribosome	None	None	Hamamoto R,et al. [66]	Chan CT, et al. [67]	[66-67]
18	SPRR1A	IC	M, cytosol	Cornified envelope, keratin filament	None	None	Lau D, et al. [68]	None	[68]
19	PLAG1	IC	N	Pericentriolar material	None	Matsuyama A , et al. [69]	Declercq J, et al. [70]	None	[69-70]
20	PDCD10	IC	EC;M;cytosol; golgi apparatus	Chromosomal region	None	Non e	Lauenborg B, et al. [71]	None	[71]
21	SP140L	IC	N	Synaptic membrane	None	None	None	None	
22	POLR2H	IC	Cytosol;N	Nucleolar part, DNA-directed RNA polymerase III complex, DNA-directed RNA polymerase I complex, DNA directed RNA	None	None	None	None	

				polymerase II holoenzyme, nucleoid					
23	PPP1R13B	IC	Nucleus;M; cytosol	Centrosome	None	None	Wang Y, et al. [72]	None	[72]
24	ALG3	M	Endoplasmic reticulum	Ribosome	None	None	Not conclusive	None	
25	EXOSC4	IC	Cytosol;N; cytoskeleton	Organellar large ribosomal subunit	None	None	O'Byrne K, et al [73].	None	[73]
26	DVL3	IC	Cytosol;N	WNT signalosome, nuclear chromosome	Hasan R, et al. [74]	You XJ, et al. [75]	Zhao H, et al. [76]	None	[74-76]
27	SLC2A1/GLUT1	M, IC	M;EC;cytoskeleton	Cell cell junction, apical plasma membrane, cytoplasmic region, keratin filament	Ogane N, et al. [77]	Goos JA, et al. [78]	Wellberg EA, et al. [79]	Sohn CH, et al. [80]	[77-80]
28	SENP5	IC	nucleus	not assigned	None	None	Jin ZL, et al. [81]	None	[81]
29	ETNK2	IC	cytosol	not assigned	None	None	None	None	
30	FNDC3B	IC,M	M;endoplasmic reticulum	Endoplasmic reticulum lumen	None	None	Chen CF, et al. [82]	None	[82]

31	PIK3 R4	IC	Cytosol;endosome;cytoskeleton	Vesicle membrane, extrinsic component of membrane, endocytic vesicle, nuclear envelope, proteinaceous extracellular matrix	None	None	Not conclusive	None	
32	MCC C1	IC,S	Cytosol;mitochondrion	Mitochondrial matrix, vesicle membrane	None	None	None	None	
33	ABC C5/MRP5	IC,M	M	Not assigned	None	None	Mourskaia AA, et al. [83]	None	[83]
34	Septin9	IC,S	Cytoskeleton	Not assigned	None	Tóth K, et al [84]	Liu M, et al. [85]	None	[84-85]
35	ADIP OQ	S	EC;endoplasmic reticulum	Collagen trimer, proteinaceous extracellular matrix	None	None	Name N, et al. [86]	None	[86]
36	ENT PD1/ CD39	M	EC;M	Side of membrane	None	Cai XY, et al. [87]	Cai XY, et al. [88]	None	[87-88]

37	CORO1C	IC	M; cytoskeleton	Plasma membrane protein complex, cell substrate junction, endoplasmic reticulum subcompartment	None	None	Luan SL, et al. [89]	None	[89]
38	DCUN1D1	IC	Nucleus, cytosol	PML body	None	None	Fu W, et al. [90]	None	[90]
39	EXT1	IC,M	Golgi apparatus;endoplasmic reticulum	Anchoring junction	None	None	de Andrea CE, et al. [91]	None	[91]
40	HIST3H2A	IC	EC;N	Not available	None	None	Aihara H,et al. [92]	Jullien D, et al. [93]	[92-93]
41	KRT6A	IC	EC; N; cytosol	Intermediate filament, keratin filament, intermediate filament cytoskeleton, cornified envelope	None	None	Hu J, et al. [94]	None	[94]
42	RNF7	IC	N; cytosol	Nuclear ubiquitin	None	None	Sun Y,et al [95]	None	[95]

				ligase complex, secretory vesicle					
43	LEPR	M	M	Nuclear chromosome	Howard JM, et al. [97]	Milosevic VS,et al. [96]	Milosevic VS,et al. [96]	None	[96-97]
44	KRT16	IC	EC;N;cytoskeleton;cytosol	Intermediate filament, intermediate filament cytoskeleton, cornified envelope	None	None	Not conclusive	None	
45	SELENOT	M	Endoplasmic reticulum	Extracellular matrix	None	None	None	None	
46	JUP	IC	M; EC; N	Desmosome, apicolateral plasma membrane, intermediate filament cytoskeleton, cell cell junction	None; Downregulated [99]	Luque-García JL,et al. [98]	None	None	[98-99]
46	KRT17	IC	EC;cytoskeleton;cytosol	Intermediate filament, keratin filament cytoskeleton	None	Kim CY, et al. [100]	Hu H. et al. [101]		[100-101]
47	CEP63	IC	Cytoskeleton; cytosol	Centrosome, PML body	None	None	Not conclusive	None	

48	IFNGR1	IC,M,S	M	Side of membrane	None	Simpson JA, et al. [102]	Zhang C, et al.[103]	None	[102-103]
49	PTK2/FAK	IC	N;M;cytoskeleton;cytosol	Cell cortex, cytoplasmic region, cytoplasmic side of membrane	Miyazaki T, et al. [104]	Cance WG, et al. [105]	Kessler BE, et al. [106]	Liao X,et al. [107]	[104-107]
50	PITX2	IC	nucleus	not assigned	Zhang JX, et al. [108]	None	Huang Y, et al. [109]	None	[108-109]
51	TFG	IC	Cytosol;EC;endoplasmic reticulum	Rough endoplasmic reticulum membrane	None	None	Kim AY,et al. [110]	None	[110]
52	CLIP4	IC	Cytosol	Not assigned	None	None	Not conclusive	None	
53	PLN	M	Mitochondrion;endoplasmic reticulum;	Sarcoplasm, transporter complex, sarcoplasmic reticulum membrane, cation transporting atpase	None	None	None	None	

				complex, atpase complex, myosin filament					
54	IGF2 BP2	IC	N; cytoskeleton; cytosol	Not assigned	Barghash A,et al. [113]	Liu W, et al. [111]	Ye S, et al. [112]	None	[111-113]
55	ELF3 /ESE 1/ER T/ES X	IC	Nucleus;cytosol	Apical plasma membrane	None	None	Yachida S, et al. [114]	None	[114]
56	MMP 12	S	EC	Proteinaceous extracellular matrix	Ding Y, et al. [115]	Zheng J, et al. [116]	Xu J, et al. [117]	None	[115-117]
57	MRC 1	M	M; endosome	Endosomal part, endocytic vesicle	None	None	Not conclusive	None	
58	BGN	S	EC	EC, sarcolemma, golgi lumen, lysosomal lumen, vacuolar lumen	Zhu YH, et al. [118]	Wang B, et al. [119]	Xing X, et al. [120]	None	[118-120]
59	SSFA 2	IC	N;M;cytosol	Side of membrane	None	None	Okayama A, et al. [121]	None	[121]

60	TP63	IC	Nucleus;cytosol	Chromatin, nuclear chromosome, cornified envelope	Hu H,et al. [122]	Song Y, et al. [123]	De Oliveria DH, et al. [124]	Schütz M,et al. [125]	[122-125]

Supplementary Table S4. *Immunohistochemistry (IHC) protocols for four targets proteins*

Target protein	Protein location	Antigen retrieval	Concentration	1st antibody	Company	Mono/poly-clonal	Dye
GLUT-1	membrane	Tris/EDTA (pH 9.0)	1:250 for TMA slices; 1:500 for biopsies.	Ab115730, RRID: AB_10903230	Abcam, Cambridge, UK	Monoclonal	AEC and DAB
ENTPD1	Membranous+cytoplasm	Tris/EDTA (pH 9.0)	1:1000	Ab223842	Abcam, Cambridge, UK	Monoclonal	DAB
LEPR	Mainly cytoplasm, with some nucleus staining	Citrate buffer (10mM, pH 6.0)	1:100	Ab60042, RRID:AB_943984	Abcam, Cambridge, UK	Polyclonal	DAB
LEPR	cytoplasm	Citrate buffer	1:10	Ab2143,	Abcam,	Monoclonal	DAB

		(10mM, pH 6.0)		RRID:AB_302860	Cambridge, UK		
LEPR	cytoplasm	Tris/EDTA (pH 9.0)	1:200	Sc8391, RRID:AB_627882	Santa Cruz Biotechnology, Dallas, Texas, USA	Monoclonal	DAB
IFNGR1	cytoplasm	Tris/EDTA (pH 9.0)	1:50	Sc28363, RRID:AB_673491	Santa Cruz Biotechnology, Dallas, Texas, USA	Monoclonal	DAB
IFNGR1	cytoplasm	Citrate buffer (10mM, pH 6.0)	1:100	Ab134070	Abcam, Cambridge, UK	Monoclonal	DAB

Supplemental figure

Supplementary Fig. S1 *The IHC staining intensity score of esophagus epithelial cells.* No IHC positive staining is scored as 0. Score 1 means weak IHC staining. Score 2

means moderate staining intensity. Score 3 means strong staining intensity.

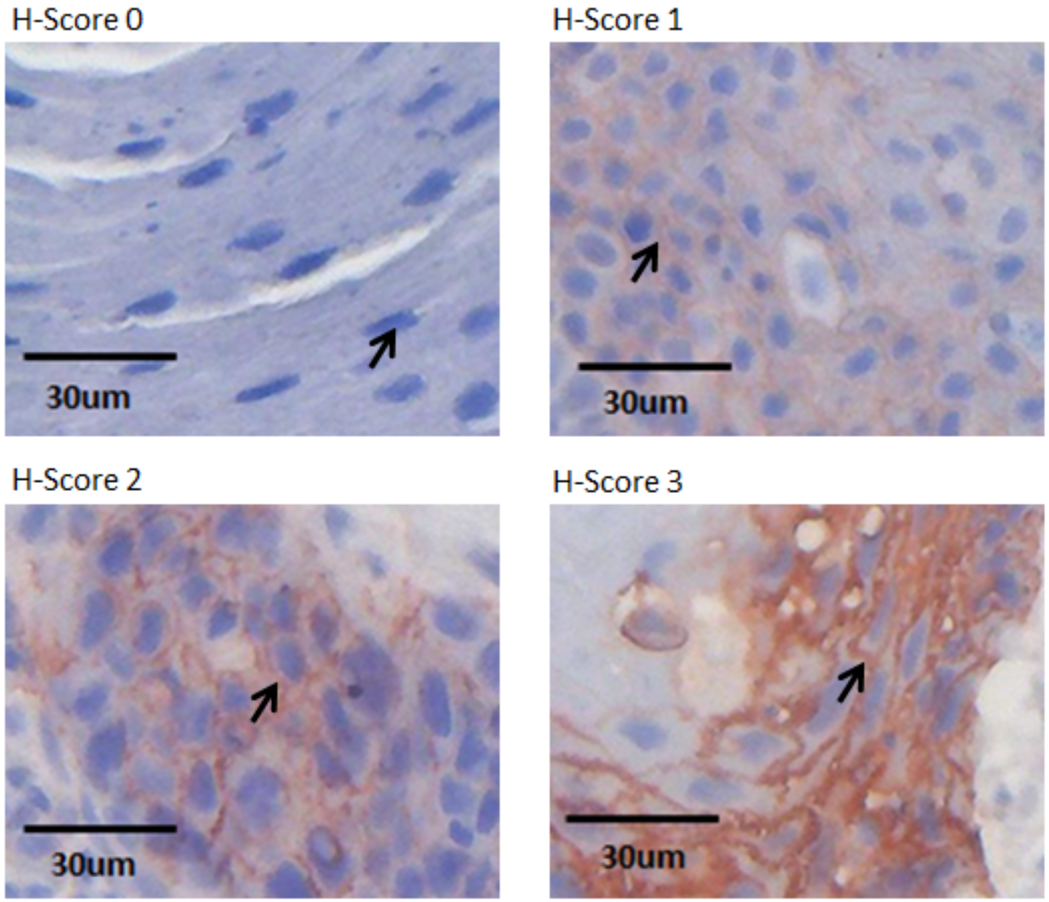

Supplement: Supplementary file 1 [file ijms-22-09270-s001.zip › Final supplementary files/Other supplementary materials-final.pdf]
